# *In Vitro* Induction of Endothelial Apoptosis of the Post-Hypoxic Blood-Brain Barrier by Isoflurane but Not by Sevoflurane and Midazolam

**DOI:** 10.1371/journal.pone.0130408

**Published:** 2015-06-19

**Authors:** Michael S. Dittmar, Walter Petermichl, Regina Lindner, Barbara Sinner, Bernhard M. Graf, Felix Schlachetzki, Michael Gruber

**Affiliations:** 1 Department of Anesthesiology, Regensburg University Medical Center, Regensburg, Germany; 2 Department of Neurology, Bezirksklinikum Regensburg, University of Regensburg, Regensburg, Germany; Julius-Maximilians-Universität Würzburg, GERMANY

## Abstract

**Background:**

The effects of anesthetics on the injured brain continue to be the subject of controversial discussion. Since isoflurane has recently been shown to induce apoptosis of cerebral endothelial cells, this study compared different anesthetic compounds regarding their potential to induce cerebro-vascular apoptosis.

**Methods:**

The *in vitro* model of the blood-brain barrier used in this study consisted of astrocyte-conditioned human umbilical vein endothelial cells (AC-HUVEC) has been used. After 24 h of deep hypoxia and reoxygenation or control treatment, AC-HUVEC were exposed to 0, 0.5, 1.0, or 2.0 times the minimum alveolar concentration of isoflurane or sevoflurane, or 0, 75, 150, or 300 nM of midazolam for 2 h. After 24 h, AC-HUVEC were harvested, and the degree of apoptosis was assessed by means of Western blots for the Bax and Bcl-2 ratio and, for controls and the highest concentration groups, terminal deoxynucleotidyl-mediated dUTP-biotin nick end labeling (TUNEL).

**Results:**

Without hypoxic pretreatment, 2.0 MAC of isoflurane slightly increased TUNEL intensity compared to control and sevoflurane, but without any significant changes in the Bax and Bcl-2 ratio. After hypoxic pretreatment, exposure to isoflurane led to a multifold increase in the Bax and Bcl-2 ratio in a dose dependent manner, which was also significantly higher than the ratio observed in the 2 MAC sevoflurane group. TUNEL intensity in the post-hypoxic 2 MAC isoflurane group was increased by a factor of 11 vs. control and by 40 vs. sevoflurane. Sevoflurane and midazolam did not significantly alter these markers of apoptosis, when compared to the control group.

**Conclusions:**

Isoflurane administered after hypoxia elevates markers of apoptosis in endothelial cells transdifferentiated to the cerebro-vascular endothelium. Endothelial apoptosis may be a previously underestimated mechanism of anesthetic neurotoxicity. Administration of high concentrations of isoflurane in experimental settings may have negative effects on the blood-brain barrier.

## Introduction

Reports on the effect of volatile anesthetics on the healthy and the injured brain are contradictory. Some authors have described neuroprotective properties via several mechanisms [1–9], whereas other publications suggest toxic effects of anesthetics on developing [10–15] or injured [16,17] neurons.

Since the pathophysiological focus on CNS damage has widened from a narrow neurocentric view towards a more holistic understanding of the complex interactions within the neurovascular unit, the cerebral endothelium has again become a target for research and therapy. Disruption of the blood-brain barrier (BBB), subsequent cerebral edema and the entry of potentially toxic blood serum ingredients, as well as the translocation of inflammatory cells are typical consequences related to cerebral endothelial dysfunction in several brain diseases such as trauma, stroke, and global cerebral hypoxia or ischemia.

Recently, we have been able to show that isoflurane has the potential to induce endothelial apoptosis in an *in vitro* model of the post-hypoxic BBB [18]. Yet, the question if different anesthetics have a different apoptogenic potential remains unanswered. In the current study, we investigated the influence of different concentrations of isoflurane, sevoflurane and midazolam with regard to their risk of inducing endothelial apoptosis, either with or without previous hypoxia. Unlike isoflurane, sevoflurane and midazolam were not associated with increased endothelial apoptosis.

## Materials and Methods

### *In vitro* model of the BBB

Human umbilical vein endothelial cells (HUVEC) were derived from the STEMMAT project [19] and were provided by the department of cardiac surgery at the Regensburg University Medical Center. The umbilical cords were obtained with approval of the ethics committee (ethics committee at the University of Regensburg No. 03/046MZ and ethics committee at the Technical University of Munich No. 797/03) and written informed consent of the patients.

The methods used in our study have been previously described in detail elsewhere [18]. In brief, primary human umbilical vein endothelial cells (HUVEC) were provided by the department of cardiac surgery at the Regensburg University Medical Center. Harvested cells were frozen in liquid nitrogen until use. Prior to the study, HUVEC were thawed and cultured up to passage five. To achieve transdifferentiation into cerebral endothelium—like cells, HUVEC were grown in 50% (vol/vol) modified endothelial cell growth medium (ECGM Provitro, Berlin, Germany) and 50% astrocyte-conditioned medium (ACM). ACM was harvested from cultures of the U-87 line (ATCC, Wesel, Germany), a glioblastoma (astrocytoma IV°) cell line. No co-culture of HUVEC and astrocytes was used, and all experiments were done with HUVEC-only cultures.

Transdifferentiation of HUVEC into cerebral endothelium-like cells was verified by measuring the transendothelial electrical resistance (TEER). Experiments were started after four days of ACM conditioning at which specific TEER values peaked above 600 Ωcm².

### Hypoxia

For hypoxia, confluent astrocyte-conditioned HUVEC (AC-HUVEC) were transferred into a BBD 6220 humidified hypoxia chamber (Thermo Scientific Heraeus, Langenselbold, Germany) at 3% O_2_ and 5% CO_2_. After 24 h of hypoxia, the flasks were placed in the normoxic incubator to allow a reoxygenation period of 2 h. The AC-HUVEC were then subjected to anesthesia or control treatment. Non-hypoxic groups were kept in the standard incubator for the same period of time.

### Anesthesia treatment

The AC-HUVEC were either treated with isoflurane, sevoflurane, or midazolam. Cells in the control group did not have any contact with either substance. Volatile anesthetic delivery was achieved by means of a modified anesthesia unit Trajan 808 (Draeger, Lübeck, Germany) in air (95%) and CO_2_ (5%). Anesthesia gas vapors for either isoflurane (Forane, Abbott India, Verna Salcette, India) or sevoflurane (Baxter Healthcare, Halle / Westfalen, Germany) were installed to add the anesthetic. The gas mixture was introduced into cell culture flasks, which were maintained at 37°C. For continuous monitoring of the gas composition, a Capnomak Ultima monitor (Datex Engstrom, Fairfield, CT, United States) was used. During midazolam and control treatment, cells were aerated by the same mode of gas supply, without adding any volatile anesthetic.

The AC-HUVEC were treated with either isoflurane or sevoflurane at concentrations of minimal alveolar concentrations (MAC) of 0.5, 1 or 2 for 2 h, or by adding midazolam (Ratiopharm GmbH, Ulm, Germany) to the culture medium at concentrations of 75, 150, or 300 nM for 2 h. One MAC was considered 1.3 vol% for isoflurane and 2.4 vol% for sevoflurane. After 2 h of treatment, the volatile anesthetic was washed out with 95% air and 5% CO_2_ for 0.5 h in the isoflurane and sevoflurane group, or removed by three washing cycles with fresh medium in the midazolam group. Subsequently, the cells were returned to the standard incubator for 24 h of recovery before we proceeded with harvest and analysis.

### Western blot analysis

The expression of the apoptosis marker Bcl-2–associated X protein (Bax) and the anti-apoptotic B-cell lymphoma protein 2 (Bcl-2) was analyzed by Western blotting (n = 3 independent experiments per group) [20,21]. The cells were harvested, lysed, and centrifuged at 8,400 g before the supernatant was removed and frozen at −80°C until analysis.

For gel electrophoresis, protein samples were diluted 3:1, and 40 μg of protein were loaded per lane onto 10% acrylamide SDS separating gels (Sigma Aldrich). We used RAW 264.7 (IP) Cell Lysate (SC- 2211, Santa Cruz Biotechnology, Heidelberg, Germany) for Bax and WEHI 231 Cell Lysate (SC- 2213, Santa Cruz) for Bcl-2 for positive control. Following electrophoresis, the separated proteins were blotted on Membrane Hybond-CExtra nitrocellulose (Amersham, Bucks, UK) at 300 mA for 60 min. The blots were then rinsed and blocked with milk powder. We used a 1:5,000 dilution of the primary antibody anti-ß-actin from mice (A5316, Sigma Aldrich), and a dilution of 1:1,000 of the primary antibody anti-Bax or anti-Bcl-2 from rabbits (Cell Signaling, Frankfurt am Main, Germany). After over-night incubation at 4°C, we added a 1:15,000 dilution of the fluorescence-labeled secondary antibodies anti-mouse and anti-rabbit, produced in donkey (700/800 IRDye, LI-COR Biosciences GmbH, Bad Homburg, Germany). The blots were incubated at room temperature in darkness for 1 h. Bax and Bcl-2 levels were normalized against ß-actin, and the Bax and Bcl-2 ratio was calculated.

### TUNEL staining and microscopy

To assess the degree of apoptosis, the terminal deoxynucleotidyl-mediated dUTP-biotin nick end labeling (TUNEL) technique was applied. AC-HUVEC were grown on cover glasses and subjected to the anesthetic treatment procedure described above (2 MAC or 300 nM only, n = 2 independent experiments per group). After the recovery period, the cells were fixed with 4% paraformaldehyde solution (Carl Roth, Karlsruhe, Germany) and stained with an In-situ Cell Death Detection Kit (Fluorescein 116847959–10 kit, Roche, Mannheim, Germany). For read-out, we took three pictures by a fluorescence microscope, allocated in a pre-defined manner, and used the mean of overall image fluorescence for further calculations.

### Statistical analysis

Repeated measurements of individual cases were averaged and were analyzed as one case to avoid inappropriate case number duplication. All values are expressed as means and ranges. Statistical analysis was done with SPSS Statistics 21.0 (IBM Corporation).

The effect of each concentration of the anesthetics on the Bax and Bcl-2 ratio was compared against control and the remaining concentration groups of the same agent by a single factor analysis of variances (ANOVA) with Dunnett-T3 post-hoc testing. Further, isoflurane and sevoflurane were compared against each other within the corresponding MAC group using the Student T test. The results were corrected for an assumed inequity of variances. Midazolam groups were not directly compared against the volatile anesthetics, since equipotency of the concentrations chosen is not warranted. Differences were considered statistically significant at P < 0.01.

TUNEL intensity of the 2.0 MAC / 300 nM groups was compared against control and between isoflurane and sevoflurane using the ANOVA with Dunnett-T3 post hoc test. For TUNEL experiments, differences were assumed to be significant at P < 0.05.

## Results

### Increased Bax and Bcl-2 ratio after hypoxia and isoflurane treatment

**Fig 1:**
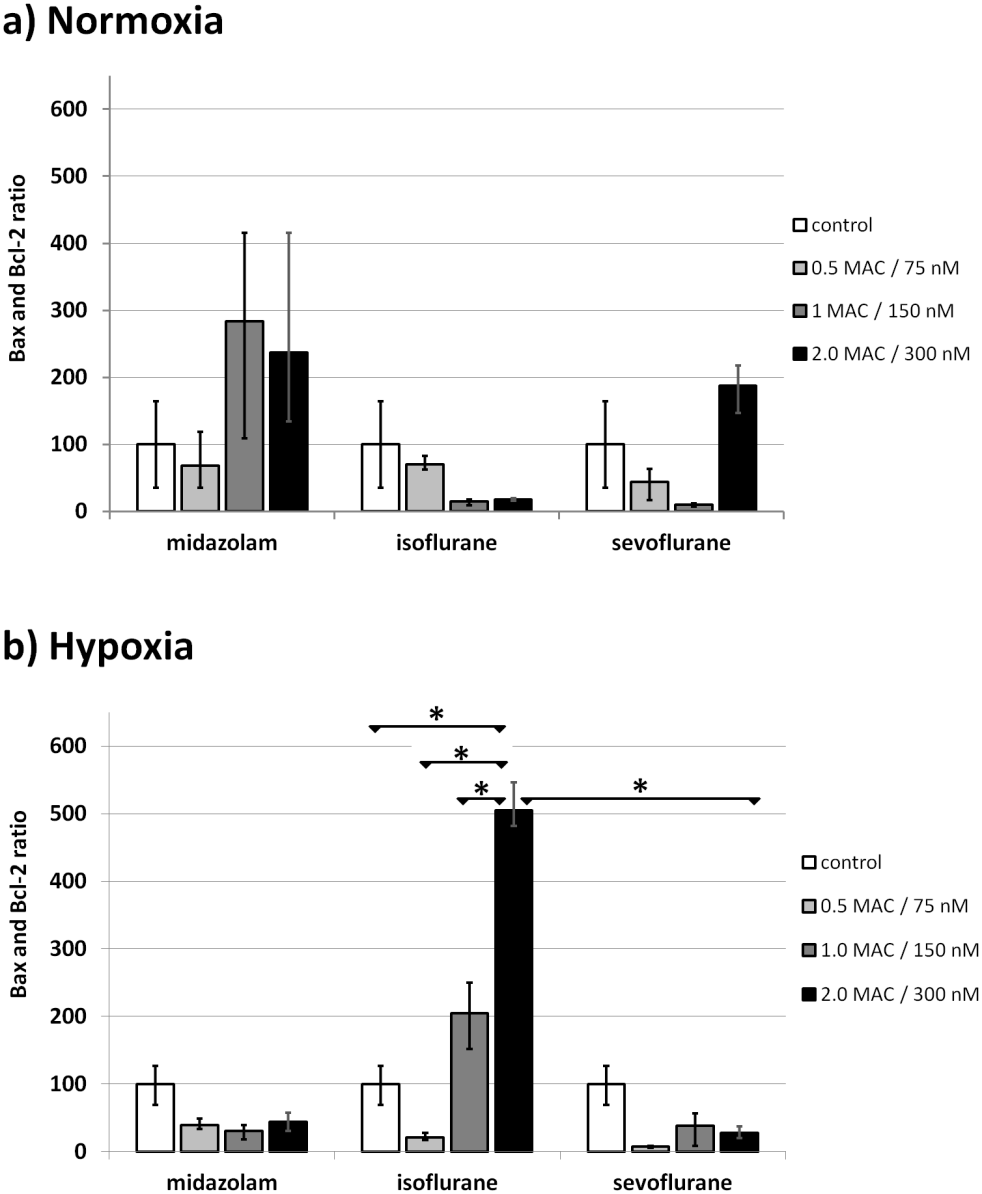
Influence of anesthetics on pro- and anti-apoptotic proteins. Bax and Bcl-2 ratio of normoxic (a) and post-hypoxic (b) AC-HUVEC after post-treatment with various concentrations of midazolam, isoflurane, and sevoflurane for 2 h, and recovery of 24 h. N = 3. * P < 0.01 (ANOVA).

Without previous hypoxia, no significant differences in the Bax and Bcl-2 ratio could be detected in relation to the tested compounds (Fig 1).

**Fig 2:**
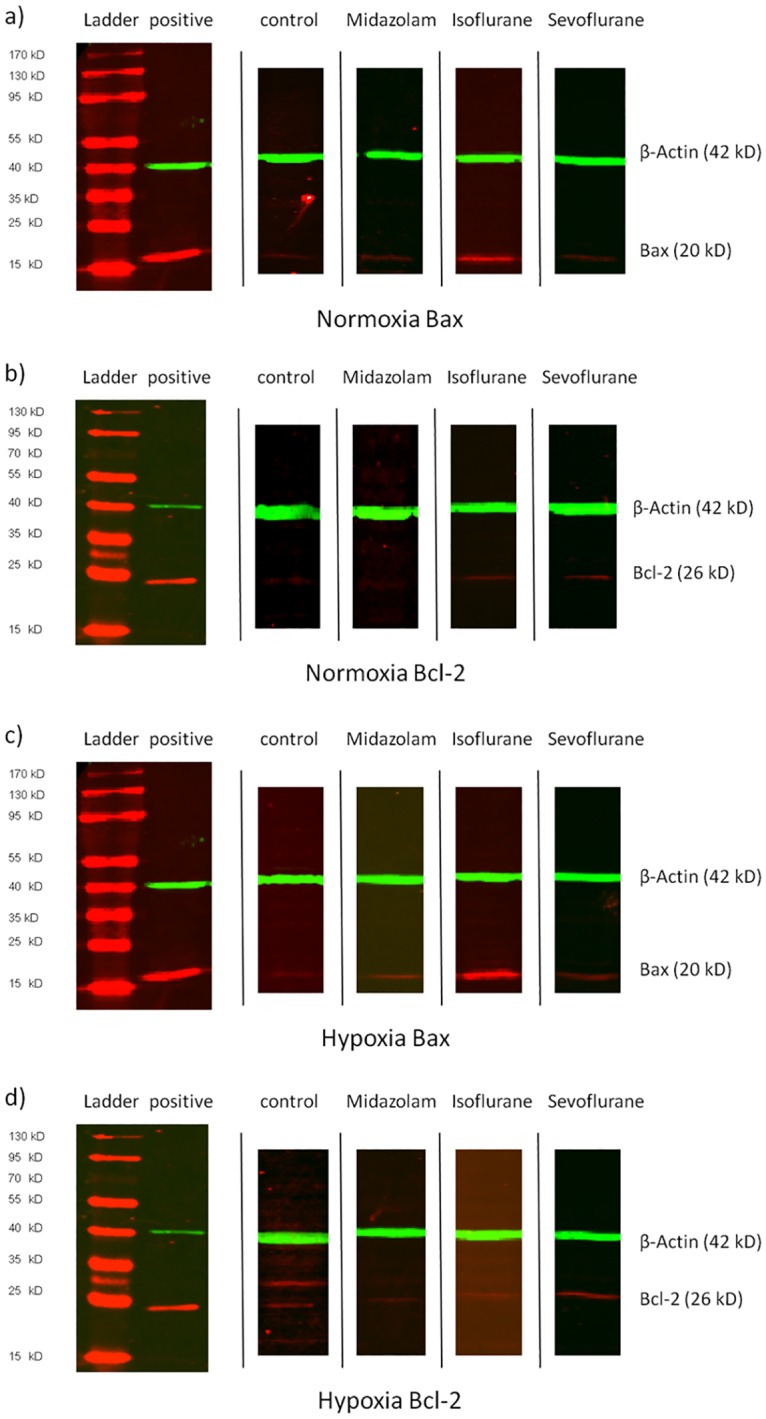
Bax and Bcl-2 Western blots. Representative Western blot clippings for Bax and Bcl-2 from normoxic (Fig 2a and 2b) and post-hypoxic (Fig 2c and 2d) AC-HUVEC harvested 24 h after treatment with 300 nM of Midazolam, 2 MAC Isoflurane, 2 MAC Sevoflurane or no anesthetic compound (control). Positive: positive control.

After 24 h of hypoxia, exposure to 2 MAC of isoflurane for 2 h followed by 24 h of reoxygenation increased the Bax and Bcl-2 ratio by a factor of 5 compared to control (P = 0.001), by a factor of 24 compared to 0.5 MAC (P = 0.005), and by a factor of 2.5 compared to 1 MAC of the same agent (P = 0.006) (Fig 1, Table 1). In addition, when compared to 2.0 MAC of sevoflurane, there was a significant 18-fold increase in the Bax and Bcl-2 ratio (P = 0.001) (Fig 1, Table 1). Representative Western blot clippings for the 2 MAC and 300 nM groups are shown in Fig 2.

**Table 1:** Summary of results.

Anesthetic compound	Concentration	No hypoxia		Hypoxia	
		Bax and Bcl-2 ratio (range)	TUNEL intensity (range)	Bax and Bcl-2 ratio (range)	TUNEL intensity (range)
Control	-	**5.80** (2.03–9.15)	**4,322** (4,237–4,407)	**8.98** (6.20–11.38)	**3,632** (3,213–4,052)
Midazolam	75 nM	**3.97** (2.02–6.90)	-	**3.52** (2.95–4.36)	-
	150 nM	**16.45** (6.31–24.12)	-	**2.69** (1.66–3.52)	-
	300 nM	**13.76** (7.76–24.15)	**1,117** (882–1,353)	**3.99** (2.71–5.16)	**3,007** (2,902–3,111)
Isoflurane	0.5 MAC	**4.08** (3.60–4.84)	-	**1.86** (1.54–2.48)	-
	1.0 MAC	**0.85** (0.55–1.06)	-	**18.37** (13.68–22.43)	-
	2.0 MAC	**1.02** (0.91–1.16)	**11,059** * (10,957–11,160)	**45.31** ** (43.27–49.07)	**38,592** * (37,378–39,805)
Sevoflurane	0.5 MAC	**2.54** (0.99–3.64)	-	**0.62** (0.41–0.74)	-
	1.0 MAC	**0.58** (0.38–0.69)	-	**3.42** (0.74–5.05)	-
	2.0 MAC	**10.89** (8.52–12.65)	**1,665** (1,261–2,069)	**2.51** (1.84–3.30)	**959** (733–1,184)

Results for markers of apoptosis (Bax and Bcl-2 ratio, TUNEL intensity) in the treatment groups with and without previous hypoxia. Values are means and range. Midazolam groups have not been compared against isoflurane and sevoflurane groups.

* P < 0.05 vs. control and sevoflurane.

** P < 0.01 vs. control, isoflurane 0.5 MAC, isoflurane 1 MAC, and sevoflurane 2 MAC.

### Increased TUNEL intensity after isoflurane treatment

**Fig 3:**
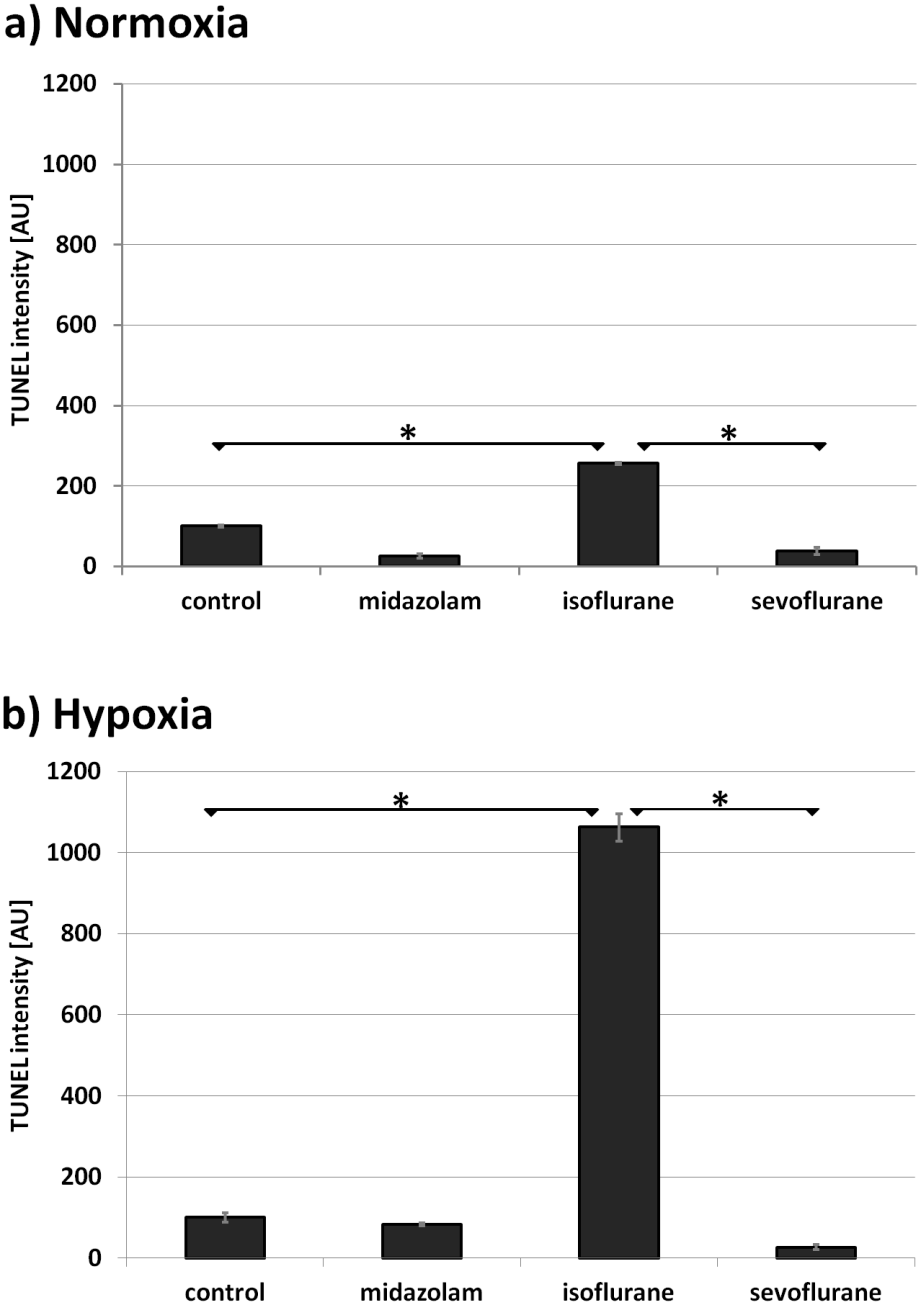
Anesthetics and endothelial apoptosis measured by TUNEL. TUNEL intensity of AC-HUVEC without (a) and with hypoxia (b) 24 h after post-treatment with 300 nM of midazolam, 2 MAC of isoflurane, or 2 MAC of sevoflurane. * P < 0.05 (N = 2, ANOVA).

Cells treated with isoflurane but not hypoxia showed a significant increase in TUNEL intensity with values exceeding those of control and sevoflurane by a factor of 2.6 (P = 0.001) and 6.6 respectively (P = 0.043) (Fig 3a, Table 1).

The TUNEL response was heavily augmented in post-hypoxic cells. In this case, HUVEC treated with isoflurane showed increased fluorescence levels by a factor of 10.6 vs. control (P = 0.026) and 40.3 vs. sevoflurane (P = 0.036) (Fig 3b, Table 1). Sevoflurane and midazolam did not significantly alter TUNEL intensity when compared to control values.

**Fig 4:**
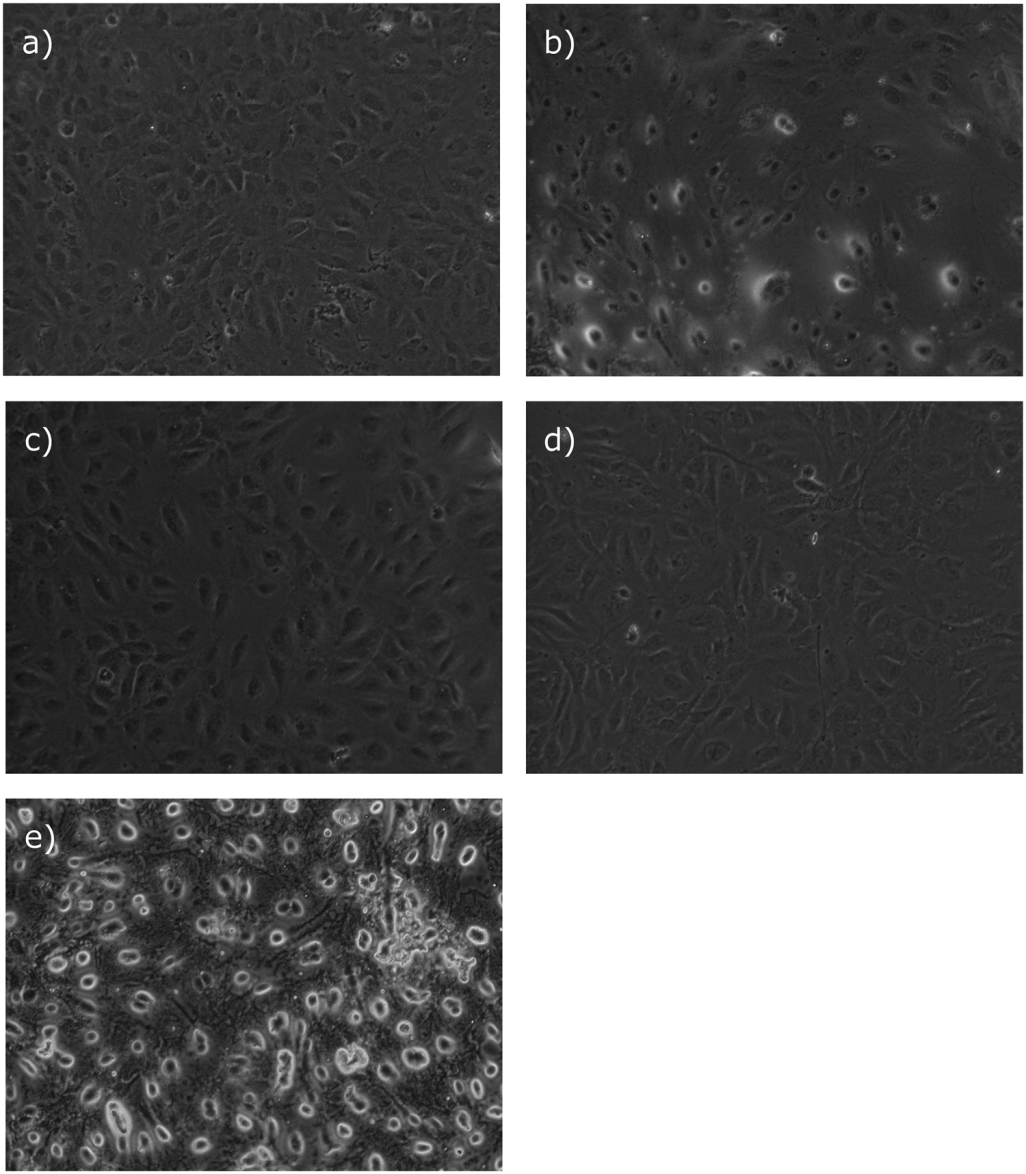
TUNEL fluorescence microscopy. Representative TUNEL fluorescence labeled light microscopy images of AC-HUVEC. Images were taken after the experimental sequence of 24 h of hypoxia, 2 h of reoxygenation, treatment with 300 nM midazolam (a), 2 MAC isoflurane (b), 2 MAC sevoflurane (c), or no anesthetic treatment (d) for 2 h, and a 24 h recovery period. Part (d) shows the positive control.

Representative fluorescence microscopy images with TUNEL labeling are shown in Fig 4.

## Discussion

In this study we showed that post-hypoxic exposure to high concentrations of isoflurane—but not sevoflurane or midazolam—increases the Bax and Bcl-2 ratio as well as TUNEL intensity, which represent markers of endothelial apoptosis, of the post-hypoxic blood-brain barrier in an *in vitro* primary endothelial cell culture model. Further, TUNEL intensity was increased after treatment with high concentrations of isoflurane without previous hypoxia.

### Evidence on anesthesia induced brain damage is conflicting

Numerous studies have addressed the influence of anesthetics on brain damage after cerebral ischemia [1–18,22,23]. However, the heterogeneity of study protocols, experimental models used, treatment regimens, and study endpoints led to inconsistent conclusions on whether anesthetics exert a beneficial or a detrimental effect in the context of acute stroke.

### BBB opening is a part of stroke pathology

In animal models of experimental transient focal cerebral ischemia, the BBB undergoes a biphasic opening with an early peak after 4 to 6 h, an intermittent partial closure of the barrier after approximately 24 h, and a second, long-lasting peak beginning approximately 48 h after the incident [24–26]. These findings have been recently confirmed in a combined *in vitro* and *in vivo* study [27]. In humans, BBB opening can be observed from 3 h to 7 d after the incident [28] and is a key factor for death due to malignant brain infarction.

### Anesthetics are associated with further BBB opening

Several recent studies have reported a disruption of the BBB or increase in brain edema, or both, after the administration of isoflurane in an *in vitro* model of the BBB [18], in healthy cats [29], in rats after transient focal cerebral ischemia [16,26], and in a mouse model of traumatic brain injury [30]. In contrast, isoflurane also has been described to prevent BBB opening in a mouse model of subarachnoid hemorrhage [2] and cerebral edema in rats subjected to middle cerebral artery occlusion (MCAO) [7].

The disruption of tight junctions has been proposed as an underlying mechanism for BBB opening after administration of isoflurane [30]. Isoflurane may therefore enhance disease-specific tight junction disturbances that are known to play a role in cerebral ischemia [31]. Oxidative stress is also of importance in this respect [16,29].

In animal models, surgery under general anesthesia with isoflurane has led to an inflammation-mediated opening of the BBB [32,33]. Sevoflurane has been associated with post-surgical BBB disruption [34], but also with a reduction of cerebral edema in combination with upregulation of Bcl-2 and downregulation of Bax in a rat model of MCAO [8]. Propofol might exert a beneficial post-conditioning effect on the post-hypoxic BBB [35]. Halothane, on the other hand, has been associated with a higher degree of late BBB opening than isoflurane in an embolic stroke model in rats [36].

Recently, Krueger and coworkers have found a BBB breakdown without any evidence for tight junction impairment in an embolic model of focal cerebral ischemia in rats [37]. Instead, trans-endothelial trafficking of tracer substances combined with endothelial degradation was seen. Notably, these animals were anesthetized with isoflurane. Thus, other mechanisms than changes of the tight junction complex may be involved in anesthesia-associated BBB opening.

### Isoflurane may cause endothelial apoptosis

In our present study, exposure to 2.0 MAC of isoflurane markedly increased TUNEL intensity, particularly after previous sustained hypoxia. Additionally, after hypoxic pretreatment, exposure to isoflurane increased the Bax and Bcl-2 ratio in a dose dependent fashion. These findings are consistent with the induction of endothelial apoptosis due to isoflurane treatment of the post-hypoxic endothelium. In a previous study, these findings were associated with morphological disintegration of the endothelial cells [18]. These findings support the thesis, that endothelial apoptosis might be a previously unrecognized mechanism of isoflurane neurotoxicity.

The adverse effects of isoflurane on endothelial cells were not present after treatment with equipotent concentrations of sevoflurane. Midazolam, applied in concentrations capable of inducing neuronal apoptosis in a hippocampal cell culture model [15], did also not negatively affect endothelial cells. Thus, endothelial toxicity may be a specific effect of isoflurane.

Instead, sevoflurane and midazolam treatment led to small decreases in the Bax and Bcl-2 ratio or TUNEL intensity, which were not statistically significant due to the relatively stringent levels of significance applied in this study. However, these study results do not contradict the possibility that midazolam, sevoflurane, or low doses of isoflurane might have protective effects on the blood-brain barrier as suggested elsewhere [2,7,8].

Isoflurane has been shown to induce neuronal apoptosis by activating the mitochondrial pathway of apoptosis via reactive oxygen species and by decreasing Bcl-2 protein expression [38,39]. This decrease was associated with an increase in Bax [38], as seen in our study. Desflurane, on the other hand, did not lead to apoptosis of neuronal cells.

### Limitations of the study

The main drawback of this study is its rather small case number. We chose a case number of n = 3 for Western blot and n = 2 for TUNEL experiments because we expected only changes by a multiple of the control value to be relevant for the conclusion of the study. Such large changes can be reliably detected with the small case number used here. Notably, the experiments for Western blotting and the TUNEL part of the study were conducted independently whereas the results are in agreement with each other. This notion further supports the validity of the results.

Isoflurane affected the BBB predominantly at a concentration of 2.0 MAC, which is a dose exerting that usually used in clinical combination anesthesia. Thus, the experimental findings of this study cannot be transferred directly to clinical patient care situations. However, if in animal experiments isoflurane is used as a single anesthetic for small surgical procedures, doses exceeding 1 MAC are often necessary.

The study’s conclusion is further limited by the restrictions of the single cell, trans-differentiated endothelial model of the BBB used for these observations. Experimental results of anesthetic effects on the BBB may differ according to the *in vitro* model used [30]. Endothelial injury can be further augmented by combined oxygen and glucose deprivation [27]. Additionally, factors derived from dying glia and neuronal cells as well as degradation of the basal membrane can further augment anesthetic effects on the neurovascular unit. Thus, further *in vivo* studies are necessary to verify the findings.

## Conclusions

According to the results of this study, treatment with isoflurane may exert a negative effect on the BBB in animal models of CNS pathologies, which might lead to artifacts. Although the use of isoflurane has still been advocated in recent publications [40]—at least in studies focusing on the BBB-, replacing isoflurane by sevoflurane or desflurane may seem preferable. Further, our findings might be interpreted as preliminary hints that patients at risk of brain edema, particularly after focal or global cerebral ischemia, might be adversely affected be the treatment with high concentrations of isoflurane. Thus, the safety profile of isoflurane should be clinically reassessed in this patient population.

In summary, the presented study provides additional evidence that the administration of isoflurane at high concentrations may exert adverse effects on the blood-brain barrier in acute brain injury. The study also shows that this effect is obviously not imminent to the same extent to all (volatile) anesthetics. Endothelial damage might be a previously unrecognized mechanism of neurotoxicity in anesthesiological pharmacology. Such damage should be considered when deciding on the use of anesthetic compounds in experimental brain research as well as in clinical neuroanesthesia.
